# Clinical characteristics and quality care indicators of pediatric stroke in a referral center of Colombia: eleven-year experience (pediastroke)

**DOI:** 10.3389/fneur.2024.1456134

**Published:** 2024-12-06

**Authors:** Valeria Valencia-Cifuentes, Natalia Llanos-Leyton, Maria Camila Gómez-Ayala, Camila Ariza-Insignares, Julian Alejandro Rivillas, Ana María Granados-Sánchez, Juan Camilo Márquez, Laura Galvis-Blanco, Santiago Cruz-Zamorano, Juan Fernando Gómez-Castro, Rubén Eduardo Lasso, Luis Miguel Rámirez-Muñoz, Juan Manuel Castro-Varela, Paula Andrea Benavides-Llano, Pablo Amaya

**Affiliations:** ^1^Servicio de Neurología, Fundación Valle del Lili, Cali, Colombia; ^2^Departamento de Ciencias Clínicas, Universidad Icesi, Cali, Colombia; ^3^Centro de Investigaciones Clínicas, Fundación Valle del Lili, Cali, Colombia; ^4^Servicio de Medicina Materno-Infantil, Fundación Valle del Lili, Cali, Colombia; ^5^Departamento de Salud Pública y Medicina Comunitaria, Universidad Icesi, Cali, Colombia; ^6^Servicio de Radiología, Fundación Valle del Lili, Cali, Colombia; ^7^Servicio de Emergencias Pediátricas, Fundación Valle del Lili, Cali, Colombia; ^8^Servicio de Neurología Pediátrica, Fundación Valle del Lili, Cali, Colombia; ^9^Unidad de Cuidados Intensivos Pediátricos, Fundación Valle del Lili, Cali, Colombia

**Keywords:** stroke, pediatrics, cerebral ischemia, cerebral hemorrhage, mortality, quality indicators

## Abstract

**Objective:**

This study aims to describe clinical variables and quality care indicators in pediatric stroke management at a high-complexity pediatric care center in Latin America.

**Methods:**

Retrospective study of patients with stroke, aged 2–18 years from 2011 to 2021. The principal outcomes were the mRs and mortality. Differences between groups were assessed using Fisher’s exact test and the Mann–Whitney U test. We used logistic regression to explore the association between characteristics reported as relevant in literature and mortality.

**Results:**

One hundred thirty six patients included, with a median age of 11 years, 54% were male. 47% were hemorrhagic strokes, followed by ischemic strokes at 39%. One-third of the cases presented in hospital. 51% of the patients had no prior medical history. The most common symptoms were altered consciousness, headache, and hemiparesis. The median door-to-image time was 123 min. The most frequent etiologies in ischemic stroke were arteriopathies and cardiac pathology, while vascular malformation and coagulopathies were predominant in hemorrhagic stroke. No patient received reperfusion therapy. At discharge, 48% of patients had a favorable mRs. The mortality rate was 21%. Patients with in-hospital stroke have approximately 7.37 times the odds of dying compared to those with out-of-hospital stroke. Patients with hemorrhagic stroke have approximately 7.46 times the odds of dying compared to those with ischemic stroke.

**Conclusion:**

Significant gaps exist in the epidemiology and quality indicators of pediatric stroke care compared to adult protocols. Implementing a “Pediatric code stroke” protocol and conducting prospective studies are crucial for improving pediatric stroke care and outcomes.

## 1 Introduction

Pediatric stroke, although rare, garners significant attention within the medical community due to its substantial impact on morbidity and mortality in children (1, 2). Stroke in children includes hemorrhagic, ischemic, mixed stroke, TIA (Transient ischemic attack) and cerebral venous thrombosis. Hemorrhagic stroke constitutes nearly half of all strokes in children, compared to only 15% in adults (3, 4). The true global incidence of pediatric stroke remains uncertain, mainly due to variations in diagnostic coding, limited data sources, and under diagnosis resulting from divergent case definitions (5).

The incidence of pediatric stroke has been estimated to be around 2 cases per 100,000 person-years in children (5), displaying a U-shaped trend with an initial peak in infants under 1 year old and a secondary peak during adolescence (6, 7). Despite the low incidence, pediatric stroke ranks among the top 10 causes of death in children (8, 9). Previous literature indicates that males have a higher risk of mortality from pediatric stroke compared to females, with the highest death rates observed in the 15–19 age group. Hemorrhagic stroke, in particular, has a significantly higher mortality rate than ischemic stroke (8).

Between 1990 and 2013, the prevalence of pediatric stroke increased by 35% worldwide, partly due to advancements in diagnostic techniques and heightened awareness leading to improved case recognition (8). In Colombia, national-level studies are scarce, highlighting the need for local reports that provide detailed characterizations of the presentation and management of pediatric stroke.

The objective of this study is to describe the clinical variables and selected quality care indicators in stroke management and outcomes. The study was conducted at a high-complexity pediatric care reference center in Latin America.

## 2 Methods

### 2.1 Study design and participants

We conducted a retrospective study by reviewing medical records at Fundación Valle del Lili, Cali, Colombia. The study included patients from January 1, 2011, to December 31, 2021, who met the following criteria: aged between 2 and 18 years, diagnosed with ischemic, hemorrhagic, mixed stroke, TIA, or cerebral venous thrombosis, confirmed by computed tomography (CT) or magnetic resonance imaging (MRI), and hospitalized within the first 7 days of symptom onset. Patients were excluded if they had received medical or surgical treatment related to the stroke at another institution or were referred elsewhere before completing all diagnostic studies and treatment. Additionally, patients under 2 years old were excluded due to the higher incidence of stroke and different etiologies in this age group.

### 2.2 Data collection

The statistics department of Fundación Valle del Lili was requested to provide medical records of patients under 18 years diagnosed with stroke, as identified by the relevant ICD-10 codes (see Supplementary material S1). Researchers reviewed these medical records and extracted information from patients who met the selection criteria. Additionally, all imaging studies conducted during hospitalization were reviewed to extract relevant variables.

The type of stroke was classified as hemorrhagic, ischemic, mixed, TIA, or cerebral venous thrombosis based on imaging findings. Specific characteristics, such as etiology and imaging findings typical of each type of stroke were documented. Mixed stroke was defined as the simultaneous occurrence of intracerebral hemorrhage and cerebral infarction. Door-to-image time was calculated as the minutes elapsed between triage admission and the appearance of the image in the hospital system. For patients whose stroke occurred during hospitalization for another reason, length of stay was calculated from their initial hospital admission. The door-to-imaging time for in-hospital stroke patients was not calculated due to difficulties in measuring the time between the image request and the challenge of determining the exact moment of symptom onset.

The primary outcomes were in-hospital mortality, modified Rankin scale at discharge and post-discharge sequelae.

### 2.3 Statistical analysis

Before conducting the analyses, a quality assessment of the database was performed. Frequencies and proportions were calculated for categorical variables, and medians and interquartile range (IQR) for continuous variables, since the Shapiro–Wilk test indicated that none followed a normal distribution.

To compare distribution of variables between in-hospital and out-of-hospital events, we used Fisher’s exact test for categorical variables and Mann–Whitney U test for continuous variables. Additionally, we used logistic regression to explore the association between characteristics reported as relevant in literature and mortality. A *p* value of <0.05 was considered statistically significant. All statistical analyses were performed using R Studio (version 4.3.1).

Ethics approval was granted by the local IRB #1860.

## 3 Results

### 3.1 Baseline characteristics

**Figure 1:**
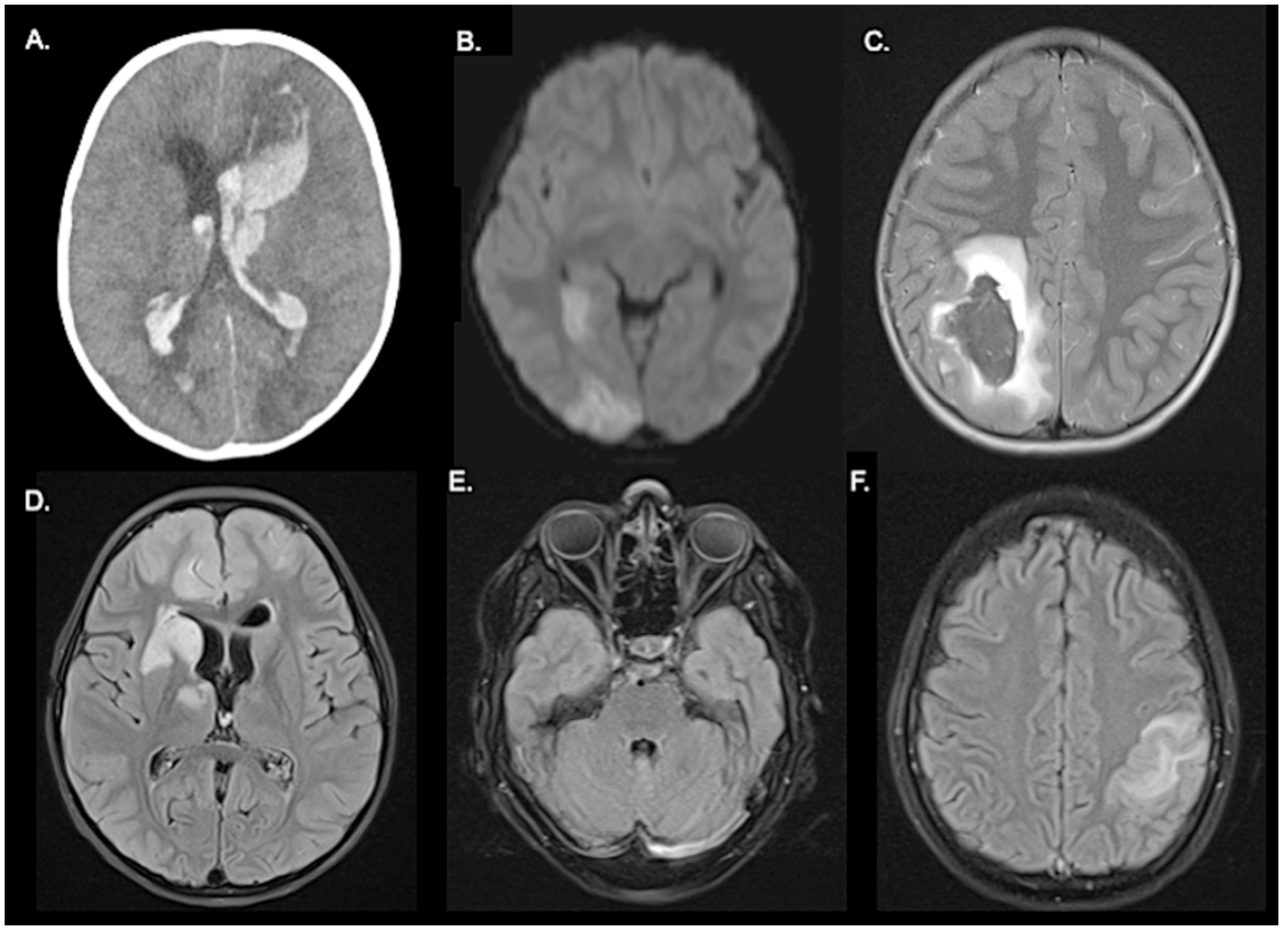
Case examples illustrating different types of stroke. Patient 1: Cranial CT **(A)** shows an intraparenchymal hemorrhage in the left frontal lobe with intraventricular drainage. Patient 2: Presented with a mixed stroke. In b1000 images **(B)**, areas of diffusion restriction are visible, involving the right posterior cerebral artery territory. T2 images **(C)** show an intraparenchymal hematoma in the right parietal lobe. Patient 3: FLAIR images **(D)** show hyperintense areas involving the right basal ganglia and frontal lobes. Patient 4: cerebral venous thrombosis: FLAIR images show thrombosis of the left transverse sinus **(E)** and a venous cortical infarct in the left cerebral hemisphere **(F)**.

A total of 136 patients met the inclusion criteria, with a median age of 11 years (IQR 6–14), of which 54% (74) were male. Nearly one-third (44) of the patients were from rural areas, and 35% (47) experienced stroke symptoms during hospitalization for another cause. 62% patients were transferred from other institutions to receive care at our hospital. Hemorrhagic stroke was the most prevalent type, accounting for 47% (64) of cases, followed by ischemic stroke at 39% (53), mixed strokes at 9% (12), and cerebral venous thrombosis at 5% (7), there were no TIA in the included sample. Additional baseline characteristics are provided in Table 1. Figure 1 shows neuroimages from cases with the different types of strokes.

**Table 1:** Characteristics of pediatric patients admitted due to acute stroke in FVL, between 2011 and 2021 (*n* = 136).

Baseline characteristics			
Age (years) (Median, IQR)		11 (6–14)	
Sex (*n*, %)	Boys	74	54%
	Girls	62	46%
Origin (*n*, %)	Urban	78	57%
	Rural	44	32%
	Metropolitan area	14	10%
Place of event (*n*, %)	Out-of-hospital	89	65%
	In-hospital	47	35%
Previous comorbidities (*n*, %)	None	70	51%
	Heart disease	23	17%
	Hemoglobinopathy	10	7%
	Malignancy	9	7%
	Previous stroke	8	6%
	Intracranial tumor	2	1%
	Thyroid disease	2	1%
	Arterial hypertension	1	1%
	Autoimmune disease	1	1%
	Diabetes	1	1%
Event characteristics			
Time since symptom onset (hours) (Median, IQR)		26 (9–96)	
Type of stroke (*n*, %)	Hemorrhagic	64	47%
	Ischemic	53	39%
	Mixed	12	9%
	Sinovenous thrombosis	7	5%
Initial neuroimaging (*n*, %)	CT	89	65%
	MRI	28	21%
	Angiography (CT,MRI, arteriography)	12	9%
Stroke etiology			
Hemorrhagic (*n* = 76)	Vascular malformation	24	32%
	Coagulopathy	15	20%
	Vasculitis	13	17%
	Aneurysm	7	9%
	Tumor	5	7%
	Arterial hypertension	3	4%
	Indeterminate	1	1%
	Others	8	11%
Ischemic (*n* = 65)	Arteriopathies	19	29%
	Heart disease	16	25%
	Malignancy	5	8%
	Sickle cell anemia	5	8%
	Hereditary thrombophilia	3	5%
	Acquired thrombophilia	2	3%
	Rheumatologic disease	2	3%
	Inborn errors of metabolism	1	2%
	Indeterminate	12	18%
Sinovenous thrombosis (*n* = 7)	Malignancy	3	43%
	Infection	1	14%
	Hematological disorders	1	14%
	Indeterminate	2	29%

For stroke etiology, the ‘n’ in hemorrhagic and ischemic stroke includes mixed cases.

Approximately half of the patients (66) had at least one risk factor, with the most common being heart diseases at 17% (23), hemoglobinopathies at 7% (10), and malignancy 7% (9). Details on the preexisting conditions are provided in Table 1. Upon admission, the initial diagnostic imaging modality was non-contrast CT scan in 65% (89) of cases, followed by non-contrast MRI in 11% (15).

### 3.2 Clinical features

According to the type of stroke, differences in clinical presentation were observed. In ischemic stroke, the most common symptoms were hemiparesis in 54% of cases (28/53), headache in 36% (19/53), language disturbances in 34% (18/53), and altered consciousness in 30% (16/53). In hemorrhagic stroke, the most frequent symptom was altered consciousness, occurring in 67% (43/64), followed by headache in 63% (40/64), nausea or vomiting in 39% (25/64), and seizures in 28% (18/64). In cases of mixed stroke, the most common symptom was also altered consciousness in 67% (8/12), followed by hemiparesis and headache, each occurring in 42% (5/12). Lastly, in patients with cerebral venous thrombosis, the most frequent symptom was headache in 71% (5/7), with nausea, vomiting, and seizures present in 43% (3/7) each, and hemiparesis in 29% (2/7). Additional manifestations are detailed in Table 2.

**Table 2:** Characteristics of different types of strokes.

		Hemorrhagic (*n* = 64)		Ischemic (*n* = 53)		Mixed (*n* = 12)		Cerebral venous thrombosis (*n* = 7)	
Clinical features									
Clinical manifestations at admission (*n*, %)	Altered state of consciousness	43	67%	16	30%	8	67%	1	14%
	Headache	40	63%	19	36%	4	33%	5	71%
	Hemiparesis	9	14%	28	53%	5	42%	2	29%
	Nausea or vomiting	25	39%	8	15%	2	17%	3	43%
	Seizures	18	28%	14	26%	0	0%	3	43%
	Language disturbance	8	13%	18	34%	2	17%	0	0%
	Gait disturbance	3	5%	5	9%	1	8%	0	0%
	Abnormal movements	1	2%	2	4%	0	0%	1	14%
	Neck pain	0	0%	1	2%	0	0%	1	14%
	Visual disturbances	0	0%	2	4%	0	0%	0	0%
	Behavioral changes	2	3%	0	0%	0	0%	0	0%
	Ataxia, vertigo, coordination disturbance	0	0%	1	2%	0	0%	0	0%
	None	1	2%	0	0%	0	0%	0	0%
	Others	16	25%	21	40%	4	33%	2	29%
Previous Comorbidities (*n*, %)	None	41	64%	24	45%	1	8%	4	57%
	Cardiopathy	5	8%	12	23%	6	50%	0	0%
	Hemoglobinopathy	4	6%	4	8%	2	17%	0	0%
	Neoplasia	1	2%	3	6%	2	17%	3	43%
	Previous stroke	2	3%	5	9%	1	8%	0	0%
	Intracranial tumor	1	2%	1	2%	0	0%	0	0%
	Thyroid disease	0	0%	2	4%	0	0%	0	0%
	Arterial Hypertension	0	0%	1	2%	0	0%	0	0%
	Autoinmune disease	0	0%	1	2%	0	0%	0	0%
	Diabetes	0	0%	1	2%	0	0%	0	0%
	Others	15	23%	15	28%	1	8%	0	0%
Systemic disease at admission (*n*, %)	None	44	69%	25	47%	4	33%	3	43%
	Sepsis	9	14%	7	13%	4	33%	1	14%
	Infection without sepsis	5	8%	7	13%	0	0%	1	14%
	Vaso-occlusive crisis	4	6%	2	4%	1	8%	0	0%
	Shock	0	0%	4	8%	2	17%	0	0%
	Acute heart failure	1	2%	1	2%	2	17%	0	0%
	Hypoxia Cardiac Arrest	0	0%	2	4%	0	0%	0	0%
	Trauma	0	0%	1	2%	0	0%	0	0%
	Others	10	16%	17	32%	8	67%	2	29%
Inhospital management									
Acute treatment (*n*, %)	Medical management	32	50%	53	100%	10	83%	7	100%
	Haematoma drainage	11	17%	NA		1	8%	NA	
	Another surgery	10	16%	NA		1	8%	NA	
	Embolization	11	17%		0%	0	0%	NA	
Intrahospital anticoagulation (*n*, %)	Yes	7	11%	13	25%	9	75%	7	100%
	No	56	88%	38	72%	2	17%	0	0%
	No information	1	2%	2	4%	1	8%	0	0%
Intrahospital antiplatelet therapy (*n*, %)	Yes	2	3%	16	30%	1	8%	1	14%
	No	61	95%	35	66%	10	83%	6	86%
	No information	1	2%	2	4%	1	8%	0	0%
Decompressive craniectomy (*n*, %)	Yes	57	89%	1	2%	1	8%	0	0%
	No	7	11%	52	98%	11	92%	7	100%
Endotracheal intubation (*n*, %)	Yes	43	67%	12	23%	4	33%	1	14%
	No	21	33%	41	77%	8	67%	6	86%
Functional outcomes									
mRs at discharge (*n*, %)	0	13	20%	4	8%	0	0%	3	43%
	1	12	19%	10	19%	0	0%	2	29%
	2	5	8%	12	23%	2	17%	2	29%
	3	3	5%	9	17%	3	25%	0	0%
	4	5	8%	10	19%	2	17%	0	0%
	5	6	9%	5	9%	0	0%	0	0%
	6	20	31%	3	6%	5	42%	0	0%

### 3.3 Type of stroke specific characteristics

For hemorrhagic stroke, the most frequent etiologies were vascular malformation in 32% (24/76) of cases, coagulopathy in 20% (15/76), vasculitis in 17% (13/76), and aneurysms 9% (7/76). Only 4% (3/76) of these patients presented with hypertensive crisis upon admission. Regarding the location of bleeding, 46% (35/76) were subarachnoid hemorrhages. Among patients with parenchymal hemorrhage, the most frequent location was lobar in 59% (45/76), followed by involvement of multiple territories in 13% (10/76), basal ganglia in 8% (6/76), and cerebellum in 7% (5/76). Bilateral hemorrhages were observed in 22% (17/76) of cases, and approximately half of the patients (51%) exhibited intraventricular drainage. The median hematoma volume at the first imaging was 22 mL (IQR 11–43).

In ischemic stroke cases, arteriopathies were the most common etiology, accounting for 29% (29/65) of patients, followed by cardiac pathology at 25% (16/65), and neoplasms and sickle cell anemia at 8% each (5/65). Among patients with ischemic stroke who underwent cerebral vessel imaging, 24% (5/21) had large vessel occlusion. The middle cerebral artery was the most frequently occluded vessel, observed in 32% (21/65) of patients, followed by the lenticulostriate arteries and the posterior cerebral artery at 11% (7/65) each, the internal carotid artery at 6%, and the posterior inferior cerebellar artery (PICA) at 5% (3/65). Involvement of multiple vascular territories was noted in 29% (19/65) of patients. Hemorrhagic transformation was evident in 18% (12/65) of cases.

Regarding patients with cerebral venous thrombosis, the primary etiology was cancer in 43% (3/7) of cases, followed by infection and hematologic disorders at 14% (1/7) each. The etiology was indeterminate in 29% (2/7) of cases.

### 3.4 Diagnosis and treatment

The door-to-image in patients who presented with extra hospital stroke was 123 min (IQR 29–351). None of the patients underwent mechanical thrombectomy or received intravenous thrombolysis, primarily due to delayed diagnosis. Regarding surgical management, hematoma drainage was performed in 17% of patients with hemorrhagic stroke and in one patient with mixed stroke, while embolization was carried out in 17% of patients with hemorrhagic stroke. Prolonged hospital stays were observed in patients with in-hospital stroke compared to those with out-of-hospital stroke. ICU stays of 12 days (IQR 4–20) versus 4 days (IQR 2–7), and hospital ward stays of 28 days (IQR 12–46) versus 10 days (IQR 7–17).

### 3.5 Outcomes

**Figure 2:**
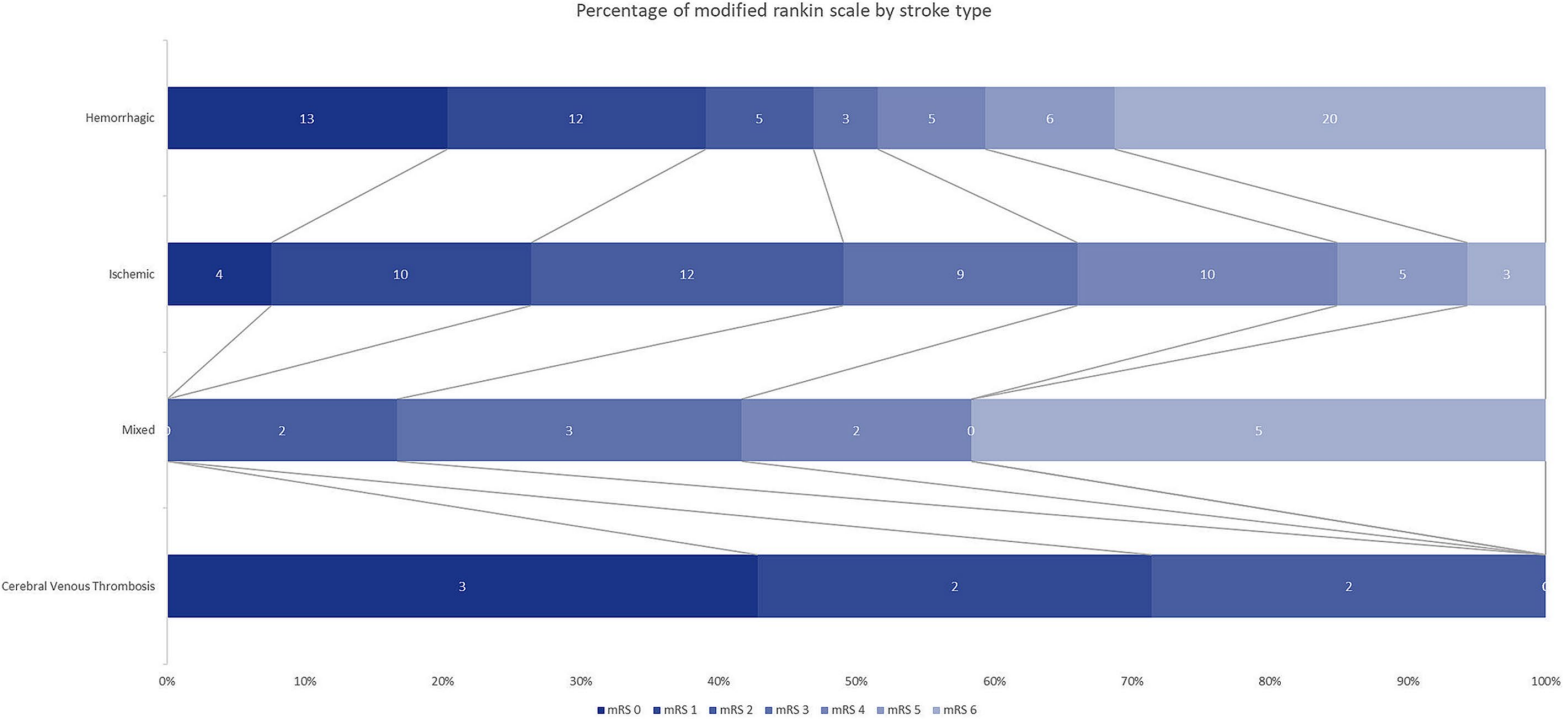
Modified Rankin Scale Scores at discharge. The distribution showed that compared with ischemic stroke, hemorrhagic stroke had a similar rate of favorable functional outcome and was associated with a higher mortality. Favorable: mRs 0–2, Unfavorable mRs 3–5, Death mRs 6.

Concerning functional outcomes at discharge, 48% of patients achieved a favorable mRS (0–2). When stratified by type of stroke, this percentage was 47% (30/64) for hemorrhagic stroke, 49% (26/53) for ischemic stroke, 17% (2/12) for mixed stroke and 100% (7/7) for CVT, the distribution of each mRS level is depicted in Figure 2. The most common sequelae at discharge were motor deficits (47%), language impairment (28%), and swallowing and gait disorders (7% each). The overall mortality rate was 21%, with a notably higher rate among patients with hemorrhagic stroke (31%) and mixed stroke (42%). Of the patients with mixed stroke, 17% (2 patients) died as a direct result of the stroke, while 25% died from underlying conditions unrelated to the stroke. Of these, one patient was on ECMO due to dilated cardiomyopathy, another was in the postoperative period following a Fontan surgery, and the third had sepsis from a fungal infection.

When comparing characteristics between groups of out-of-hospital and in-hospital stroke, significant difference were found in age (*p* = 0.014), previous comorbidity (*p* < 0.001), systemic disease at admission (*p* < 0.001) and mRs at discharge (*p* < 0.001). Additional information is provided in Table 3.

**Table 3:** Comparison of characteristics between groups of out-of-hospital and in-hospital stroke.

Characteristic	Out-of-hospital stroke (*n* = 89, 65%)	In-hospital stroke (*n* = 47, 35%)	*p* value
Sex, females (*n*, %)	41 (46%)	21 (45%)	1
Age, years (median, IQR)	11 (7–14)	8 (4–12)	**0.014**
At least one previous comorbidity (*n*, %)	31 (35%)	35 (75%)	**<0.001**
At least one systemic disease at admission (*n*, %)	16 (18%)	44 (94%)	**<0.001**
Type of stroke			
Hemorrhagic (*n*, %)	46 (72%)	18 (28%)	0.381
Ischemic (*n*, %)	33 (62%)	20 (38%)	
Mixed (*n*, %)	6 (50%)	6 (50%)	
Cerebral venous thrombosis (*n*, %)	4 (57%)	3 (43%)	
In-hospital stay			
Days in ICU (median, IQR)	4 (2–7)	12 (4–20)	**<0.001**
Days in hospitalization (median, IQR)	10 (7–17)	28 (12–46)	**<0.001**
At least one stroke sequelae (n, %)	67 (75%)	42 (89%)	0.070
mRs at discharge			
Favorable, 0–2 (*n*, %)	52 (58%)	13 (28%)	**<0.001**
Unfavorable, 3–5 (*n*, %)	29 (33%)	14 (30%)	
Death, 6 (*n*, %)	8 (9%)	20 (43%)	

IQR, interquartile range; ICU, Intensive care unit; mRs, modified Rankin scale. Bold indicates statistical significance under the two-sided *p* < 0.05 assumption.

For the logistic regression model, only the hemorrhagic stroke and ischemic stroke groups were included, as the sample sizes for the mixed and venous thrombosis groups were too small. Patients with hemorrhagic strokes had significantly higher odds of mortality compared to those with ischemic strokes (OR = 23.57, CI 95%: 5.27–153.72, *p* < 0.001), after adjusting for age, gender, previous comorbidities, systemic disease at admission and location of stroke event. Moreover, patients with intrahospital onset of stroke had over 14 times higher odds of mortality compared to those with extra-hospital onset (OR = 14.32, CI 95%: 1.81–167.25, p 0.01). Age, gender, previous comorbidities and systemic diseases at admission did not emerge as significant in this analysis. Complete data from the regression appears in Table 4.

**Table 4:** Predictors associated with mortality among pediatric patients with stroke.

Characteristic	OR	IC 95%	*p* value
Age, years	1.04	0.91–1.21	0.56
Sex, male	2.41	0.71–9.33	0.17
At least one previous comorbidity	0.59	0.13–2.20	0.45
At least one systemic disease at admission	2.10	0.22–15.75	0.48
Place of the event, in-hospital	14.32	1.81–167.25	**0.01**
Type of stroke, hemorrhagic	23.57	5.27–153.72	**<0.001**

IQR, interquartile range; ICU, Intensive care unit; mRs, modified Rankin scale. Bold indicates statistical significance under the two-sided *p* < 0.05 assumption.

## 4 Discussion

To our knowledge, this study represents one of the largest cohorts of pediatric stroke patients aged 2 to 18 years in Latin America and Colombia to date, and it is the only one presenting data on quality indicator times. Table 5 summarizes some of the previously published series worldwide. Consistent with previous reports in pediatric stroke (3) our study observed a male predominance, with 54% of the patients being male, highlighting an unexplained gender discrepancy. Hemorrhagic strokes were more prevalent in our series than reported in the pediatric literature, where they account for approximately 50% of cases (3), and in the adult literature, where they account for around 20%. In our study, hemorrhagic strokes constituted 61.9% of the cases. Mixed stroke, a rare entity, was seen in 9% of cases, characterized by the coexistence of ischemic and hemorrhagic cerebrovascular disease. Figure 1 depicts images from our series illustrating different types of strokes, including a case of mixed stroke (Figures 1B,C) presenting with an infarction in the right posterior cerebral artery territory and an intraparenchymal hematoma in the right parietal lobe.

**Table 5:** Summary of previously published series.

	Year	Country	Years	Number of patients	Age	Results
Colombia						
Factors related to the presentation of cerebrovascular attacks in children	2008	Colombia	2001–2003	65	1 month- 18y	The more compromised age group was infants (44,7%). The predominant symptom was convulsions (60%). 56,9% of all cases were ischemic events. The most common cause was infectious (20% of the cases), with a similar frequency for those who were registered as unknown cause. Mortality was 21,9%.
Characterization of hemorrhagic stroke in the pediatric population greater than 1 month of age in a high complexity hospital in Bogotá City, Colombia, from 2012 to 2017	2020	Colombia	2012–2017	55	1 month- 18y	Most patients were between 9–14 years. Intraparenchymal hematoma was the most frequent tomographic finding and hematologic causes such as leukemia, hemophilia and other bleeding and coagulation disorders were 40% of the etiology of the events. The mortality was 38% and the severe disability 12.8%.
Latin America						
Morbidity and mortality from strokes in a pediatric intensive care unit	2021	Cuba	2016–2019	45	28 days- 18y	55.5% were over 15 years old. Hemorrhagic cerebrovascular accidents were 84.4%. Conservative medical behavior in 62.2%. The CT (93.3%) was the most used image technique. Cranioencephalic traumas represented 44.4%. Mortality for years was 37.7%.
Cerebrovascular accident in a pediatric intensive care unit.	2020	Argentina	2008–2019	84	1 month- 18y	70.24% had hemorrhagic and 29.76% ischemic stroke. 60.71% were boys. The median time between symptom onset and admission to the PICU was 1 day in both groups. The mortality rate was 17.85%.
Stroke and severe disability at discharge in a pediatric population hospitalized during the period 2004–2016 in a Peruvian reference center	2018	Peru	2004–2016	140	1 month-18y	More frequent in infants (33.6%) and the male group (67.9%). Intracerebral hemorrhage was the principal type of stroke (43%). Severe disability according to PSOM scale was 32.2%.
Cerebrovascular disease in childhood. Case series	2015	Argentina	2009–2014	18	1 month-15y	Predominance of male patients and the median age of 5 years. Main clinical features: hemiparesis, seizures, headache, vomiting and sensory impairment. Most frequent type was ischemic, and the middle cerebral artery territory was the most involved. Twelve patients had no sequelae.
Worldwide						
Pediatric stroke in the northern Spanish region of Aragon: incidence, clinical characteristics, and outcomes	2021	Spain	2008–2019	21	0-15y	The mean age was 9.3 years. 8 ischemic and 13 hemorrhagic strokes. There were statistically significant differences found between ischemic and hemorrhagic strokes only in the chief complaint. None of the patients with ischemic stroke received reperfusion therapies. 42.1% had mRs > 2 at 12 months. Motor deficits were the most common sequela.
Pediatric Stroke Rates over 17 Years: Report from a Population- Based Study	2018	US	July 1, 1993–June 30, 1994, and calendar years 1999, 2005, and 2010	70	0-20y	Ischemic stroke predominated with 41 events (58.6%) compared with 27 hemorrhagic strokes (38.6%); 2 strokes were coded as unknown. Eleven children died within 30 days, yielding an all-cause case fatality rate of 15.7%. The pediatric stroke rate of 4.4 per 100.00 in the GCNK study region has not changed over 17 years.
Epidemiology and Outcomes of Arterial Ischemic Stroke in Children: The Canadian Pediatric Ischemic Stroke Registry	2017	Canada	January 1992 to December 2001	933	0-18y	National population-based study. 232 neonates, 701 older children, 55% male. The incidence was 1.72/100,000/year. Predominant clinical presentations were seizures in neonates (88%), focal deficits in older children (77%), and diffuse neurological signs (54%) in both. Neurological deficits in 60% of neonates and 70% of older children.
Cerebrovascular disease in childhood: a retrospective analysis of hospital admissions in a tertiary hospital in the community of Valencia in the last 10 years	2011	Spain	2000–2010	76	1 month-14y	44.7% had an ischemic stroke and 55.3% had a hemorrhagic one. The average age of presentation was 6.8 years. Headache was the most frequent symptom of presentation. The most frequent risk factor was vascular malformations in hemorrhagic cerebral stroke, and vascular and cardiac disorders in ischemic stroke.17% of the patients died.

Few studies have described TIA in a pediatric population. In our sample, there were no reports of TIA. In the literature, we found series in which no cases of TIA were recorded (10–12), as in ours, and series in which they were excluded due to difficulties in confirming the diagnosis (13, 14). Moreover, deVeber et al. (14) found in their series that following the initial stroke event, 12.6% of children experienced recurrent AIS or TIA. In a 3-year sample of more than 500 children hospitalized with a primary diagnosis of TIA, Adil et al. (15) found that 4.2% of the children with TIA had a secondary diagnosis of stroke within the same hospitalization. The recurrence rate of stroke after a transient ischemic attack is not negligible, so it should also be considered in pediatric patients for appropriate studies and secondary prevention when necessary. It is important to consider the diagnosis of TIA and to inquire about these types of symptoms, as more common etiologies are often considered first. When TIA is not thought of, it is not reported by the family and is not diagnosed by physicians. This highlights the importance of training pediatricians and emergency physicians to recognize and report TIA symptoms.

In contrast to the evidence observed in the adult population, the predominant clinical manifestations of pediatric hemorrhagic stroke typically do not present as focal neurological signs, which can make identification challenging (3). Our findings align with those of similar series, indicating that decreased level of consciousness and headache were the most prevalent symptoms, each presenting in half of the patients (10, 14). Hemiparesis and language alterations were the most common focal neurological symptoms, observed in 32 and 21% of cases, respectively, consistent with previous reports (12). Furthermore, seizures were present in 26% of cases, consistent with reported rates in the literature ranging from 15 to 25% (9).

In our study, approximately half of the patients (66/134) had at least one risk factor, with the most common being heart disease, followed by hemoglobinopathies and malignancy. This finding aligns with existing evidence suggesting that the development of stroke in childhood is influenced by multiple factors. These factors include a combination of inherited risk factors that predispose children to stroke and acquired factors that can precipitate a stroke occurrence (3). Hereditary risk factors encompass congenital heart disease, collagen disorders, and genetic thrombophilias, while acquired risk factors include infection, head and neck trauma, and radiation therapy for brain and head and neck cancers (7). A previous series in Colombia (16) emphasized the importance of neoplasms such as leukemias and coagulation disorders like hemophilia A among preadolescents and adolescents.

It is important to note that our study population originates from a high-complexity hospital, which leads to a higher prevalence of complex pathologies. Classic risk factors documented in adulthood, such as hypertension and diabetes mellitus, were less prevalent in our pediatric cohort, with only one patient presenting with arterial hypertension and one with diabetes mellitus. Notably, 51% of the children in our series had no previous comorbidity, which is a higher proportion compared to what has been reported in the literature (10).

Currently, hospitals in Colombia, particularly in the context of pediatric care, lack standardized protocols for pediatric stroke code management, unlike the established protocols for adults that facilitate prompt diagnosis and treatment following quality indicator of care. In our center we have developed a stroke code for children that was unfortunately implanted after our study (in 2022); mainly it is protocol for modifice nuclear magnetic resonance to prioritize specific sequences to complete the MRI in 13 min. It was developed after noticing an increase in stroke cases that had a delayed diagnosis. After the implementation of the MRI protocol we have observed an improve time to diagnostic imagen, but we will need a comparative study to document the real changes. Literature reports indicate this same significant delays, with a median time of over 20 h from symptom onset to diagnosis (17). These delays stem from challenges in distinguishing stroke from more common mimics in pediatric patients (18). Recognizing the critical importance of early diagnosis in initiating management and rehabilitation, attention must be given to the door-to-image time, which refers to the minutes from emergency department admission to the first diagnostic neuroimaging.

In adults, the ideal door-to-image time is less than 20 min (19, 20). A study conducted in Cali, Colombia (21) reported a median door-to-image time of 30 min for urban patients and 20 min for rural patients, significantly shorter than typically reported times in pediatric stroke code management. However, in our study, the median door-to-image time was 123 min (IQR 29–351). This exceeds the goal time of 60 min advocated in the literature for pediatric stroke, but aligns with findings from other studies on delayed diagnosis of pediatric stroke (17, 22).

For instance, Mallick et al. (17) reported a median door-to-image time of 253 min for arterial ischemic stroke and 72 min for hemorrhagic strokes, highlighting a significant delay in diagnosis particularly in ischemic stroke cases, which often present with more severe features. These delays pose challenges in selecting timely patient management strategies. Addressing these delays through standardized protocols and increased awareness is crucial to improving outcomes in pediatric stroke care. This issue is further underscored by the lack of standardization in neuroimaging protocols; in our series, initial imaging was a brain CT scan in 65% (89/136) of cases. Nowadays, it is recognized that performing a brain MRI with specific stroke sequences is preferable for these patients.

Among the etiologies found in our patients, the most frequent cause of hemorrhagic stroke was vascular malformations followed by coagulopathies and vasculitis. These findings are consistent with other pediatric series, which also report that intracranial vascular abnormalities are the most common etiology (2). In ischemic stroke patients, arteriopathies were the most common cause in 28% of patients, followed by cardiac diseases and neoplasms. This pattern aligns with the findings of the International Pediatric Stroke Study (23), which included approximately 676 patients and identified arteriopathies and cardiac diseases as the two leading etiologies, similar to our cohort. In that study, infections were the third most common cause, whereas in our cohort, neoplasms were the third most frequent (23). Regarding cerebral venous thrombosis, the primary cause was cancer, accounting for 43% of the cases. This finding is consistent with findings in both adult and pediatric populations.

Reperfusion therapies are less common in the pediatric population compared to adults, primarily due to delayed diagnosis in conditions where treatment efficacy is time-sensitive. In our series no one of the patients received intravenous thrombolysis primary due to delayed diagnosis: which was attributed either to a lack of symptom recognition at home or the initial misattribution of the neurological deficit to other etiologies. Additionally, thrombolytic medications are not approved for use in patients under 18 years old by the National Institute for Food and Drug Surveillance (INVIMA) in Colombia Intravenous thrombolysis, would be appropriate for children over 2 years old by the Australian Clinical Consensus Guideline in 2019 (24). However, its use remains limited in practice. Mechanical thrombectomy, which can benefit children with large vessel occlusion, is another reperfusion therapy option. Nevertheless, its application is constrained by significant inflammation often associated with the underlying pathogenesis, and the availability of appropriately sized stents is limited in many medical centers (25). In our series, no patients received reperfusion therapy due to delayed diagnosis and the absence of suitable thrombectomy devices. This underscores the critical need for improved protocols and resources to facilitate timely intervention and optimize outcomes for pediatric stroke patients.

We observed prolonged hospital and ICU stays, particularly among patients who experienced an in-hospital stroke event. The median hospital stay was 28 days (IQR 12–46), and the ICU stay was 12 days (IQR 4–20). In contrast, patients with out-of-hospital stroke had a median hospital stay of 10 days (IQR 7–17) and ICU stay of 4 days (IQR 2–7) (Table 3). Comparatively, adult patients in national series typically have shorter hospitalizations, averaging around 6 days, with ICU stays averaging 3 days. These differences can be attributed to the complexities associated with cerebrovascular events in children, including delays in diagnosis, treatment, and subsequent complications (1,4) to date, there is limited literature evaluating hospital and ICU stays specifically in children with stroke.

Currently, there is no widely adopted measure for assessing outcomes in childhood stroke, unlike the standardized stroke-specific measures developed for adults, such as the modified Rankin Scale. As a result, assessments in pediatric stroke often rely on approximations (26).

Despite efforts to develop tools for assessing stroke outcomes in children, such as the Pediatric Stroke Outcome Measure (PSOM), these tools have not achieved widespread use either clinically or in research globally (26). The PSOM, developed in 1994 at the Stroke Clinic of the Hospital for Sick Children in Toronto, Canada (26, 27), is a validated, disease-specific tool designed to assess neurological recovery in pediatric stroke patients. It includes a structured examination of 115 items across five subscales—right and left sensorimotor, language production and comprehension, and cognitive/behavioral function—providing a total score from 0 (no deficit) to 10 (maximum deficit) (27). However, only one study in the reviewed literature reported using the PSOM, underscoring its limited adoption and lack of standardization compared to the more widely used modified Rankin Scale (mRS) in adults. Consequently, we opted to use the mRS in this study.

It is estimated that at least 50% of pediatric stroke patients experience long-term neurological complications (8, 9). In our series, 53% of patients had an unfavorable outcome (mRS > 2 at discharge) (Figure 2). These findings are consistent with other series, such as the study by Lambea-Gil et al. (10) in Spain, where 42.1% of patients had modified Rankin Scale scores >2 at 12 months. Survivors of pediatric stroke face a significant risk of enduring neurological sequelae, including hemiparesis, epilepsy, cognitive impairments, and recurrent stroke episodes. Motor and language impairments were the most common sequelae observed in our patients. Consequently, the economic costs associated with childhood stroke are greater than those in adults due to a more substantial loss of disability-free life years (26).

The mortality rate in our study was 21%, which is comparable to rates reported in other series (16). Within our cohort, 71% (20/28) of deaths occurred in children with hemorrhagic stroke, even when adjusting for other variables, type of stroke has a significant relation to this outcome, consistent with previous data indicating that 64–74% of stroke-related deaths are attributed to hemorrhagic stroke (28, 29). Moreover, we found a statistically significant association between mortality and stroke onset location even adjusting for characteristics such as previous comorbidities and systemic diseases at admission. The lack of precision in the confidence intervals may be due to the small sample size, which can also result in an overestimation of the effect in the point estimates. However, supported by what the literature indicates, these variables have been associated with higher mortality, although the effect’s magnitude might be smaller.

A survey conducted among physicians at 50 pediatric hospitals across the United States aimed to assess the prevalence of stroke protocols and analyze the diagnostic and treatment components used, to identify areas of consensus (30). The findings indicated that the vast majority (92%) of pediatric hospitals have implemented stroke protocols to assist in diagnosis and treatment. However, there is still a notable lack of consensus regarding the details of these protocols, particularly concerning prehospital procedures, screening tools, and communication methods.

Drawing from the insights gained through our study, we are actively working to organize and raise awareness about the importance of establishing stroke code protocols for pediatric patients. By forming multidisciplinary teams and leveraging existing resources and networks designed for adult stroke care, we aim to enhance early detection and management of pediatric stroke. This initiative is crucial for improving patient outcomes and ensuring that children experiencing strokes receive timely and appropriate medical interventions. While there are already standardized protocols for adults, it is important to recognize that these cannot be implemented as-is for children. Therefore, understanding the specific characteristics of pediatric strokes is essential.

### 4.1 Strengths and limitations

The strengths of this study lie primarily in its position as a reference center for pediatric stroke in the southwestern region of Colombia, offering a substantial patient volume and access to advanced diagnostic imaging and treatment modalities. The inclusion of patients spanning over a decade was facilitated by the availability of digitized medical records dating back to 2011.

However, this research has several limitations that must be considered. Firstly, it is a retrospective, observational study conducted at a single center, which inherently limits the generalizability of its findings to broader populations. The rarity of pediatric stroke and the relatively small sample size further restrict the ability to conduct robust comparative analyses. Additionally, the clinical records often lacked standardized and detailed information, resulting in some data loss. There were also potential coding errors that might have affected the inclusion or exclusion of certain cases.

We did not include door-to-imaging time for in-hospital stroke patients due to the difficulty in determining the exact activation time. While door-to-imaging time is a useful quality indicator for measuring the time from suspicion of an acute neurovascular event to obtaining an initial image, which aids in ruling out other conditions aside from ischemic stroke, our study did not precisely measure the interval between clinical suspicion and confirmatory diagnostic imaging. This limitation is particularly relevant in pediatric cases, and we acknowledge the need to address this in future research.

Moreover, the study noted limited use of semi-quantitative clinical scales (such as mRS, PedNIHSS) and radiological scales (like ASPECTS) during initial evaluations and follow-ups of patients. The documentation of Rankin scale scores at discharge was not consistently recorded in the clinical records, requiring extrapolation from the last available clinical examination conducted during hospitalization.

These limitations underscore the need for caution when interpreting the findings and highlight areas for improvement in future studies, including the implementation of standardized data collection methods and more comprehensive use of clinical assessment tools.

## 5 Conclusion

We have uncovered significant gaps in the epidemiology and quality indicators of pediatric stroke care, particularly when compared to protocols established for adult stroke. The prevalence of in-hospital strokes underscores the urgency for enhanced staff training aimed at facilitating earlier detection and prompt management. Moreover, transient ischemic attacks in children often go undetected by both healthcare providers and families, highlighting the need for educational campaigns targeting both professionals and the general public. Our median door-to-image time of 123 min exceeds the recommended 60 min for pediatric stroke and falls roughly in the middle of the times reported in international studies, which range from 72 to 253 min for pediatric cases, though it remains longer than the adult benchmark of less than 20 min.

Moving forward, there is a pressing need for the development and widespread implementation of standardized protocols for pediatric stroke management, such as a dedicated ‘Pediatric Code Stroke.’ These protocols would facilitate faster diagnosis and treatment initiation across healthcare systems, ensuring timely care for pediatric stroke patients. Furthermore, future prospective studies using comprehensive assessment tools, like the PEDNIHSS (Pediatric National Institutes of Health Stroke Scale), are necessary to generate more robust, generalizable data on pediatric stroke outcomes. These initiatives are vital not only for improving care at an institutional level but for enhancing outcomes and shaping clinical guidelines globally for children affected by stroke.

## Data Availability

The raw data supporting the conclusions of this article will be made available by the authors, without undue reservation.
