# Supplementary material for: Clinical characteristics and quality care indicators of pediatric stroke in a referral center of Colombia: eleven-year experience (pediastroke)
Source: Front Neurol. 2024 Dec 6;15:1456134. doi: 10.3389/fneur.2024.1456134 (PMC11659138; doi:10.3389/fneur.2024.1456134)
Supplement: Supplementary file 1 [file Table_1.docx]

**Supplementary materials**

*CIE-10 codes included:*

| I693 | Sequelae of cerebral infarction |
| --- | --- |
| I638 | Other Cerebral Infarctions |
| I698 | Sequelae of other cerebrovascular diseases and those not otherwise specified |
| I635 | Cerebral infarction due to unspecified occlusion or stenosis of cerebral arteries |
| G458 | Other Transient Cerebral Ischemia and Related Syndromes |
| G459 | Transient cerebral ischemia, no other specification |
| I630 | Cerebral infarction due to thrombosis of precerebral arteries |
| I633 | Cerebral infarction due to cerebral artery thrombosis |
| I694 | Sequelae of cerebral vascular accident, not specified as hemorrhagic or ischemic |
| I679 | Cerebrovascular disease, unspecified |
| I64X | Acute stroke, not specified as hemorrhagic or ischemic |
| G802 | Childhood Hemiplegia |
| G81 | Hemiplegia |
| G810 | Flaccid Hemiplegia |
| G811 | Spastic hemiplegia |
| G819 | Hemiplegia, unspecified |
| I828 | Embolism and thrombosis of other specified veins |
| I677 | Cerebral arteritis, not elsewhere classified |
| I669 | Occlusion and stenosis of unspecified cerebral artery |
| I636 | Cerebral infarction due to cerebral vein thrombosis, non-pyogenic |
| G08X | Intracranial and intraspinal phlebitis and thrombophlebitis |
